# Sickness and Healing and the Evolutionary Foundations of Mind and Minding**

**DOI:** 10.4103/0973-1229.77433

**Published:** 2011

**Authors:** Fabrega Horacio

**Affiliations:** * *Professor of Psychiatry and Anthropology, University of Pittsburgh School of Medicine, Western Psychiatric Institute and Clinic, 3811 Ohara Street, Pittsburgh, PA, 15216, USA.*; ** *Revised and peer reviewed version of the paper for an International Seminar on Mind, Brain, and Consciousness, Thane College Campus, Thane, India, January 13–15, 2010.*

**Keywords:** *Disease*, *Evolutionary medicine*, *Pongid/hominin split*, *Human consciousness*

## Abstract

Disease represents a principal tentacle of natural selection and a staple theme of evolutionary medicine. However, it is through a small portal of entry and a very long lineage that disease as sickness entered behavioural spaces and human consciousness. This has a long evolutionary history. Anyone interested in the origins of medicine and psychiatry as social institution has to start with analysis of how mind and body were conceptualised and played out behaviourally following the pongid/hominin split and thereafter. The early evolution of medicine provides a template for clarifying elemental characteristics of mind and minding. Sickness and healing in chimpanzees represents an early manifestation of (ethno) medicine, termed a behavioural tradition, which is found played out in routines of helping, caring, and healing as well as other social behaviours. Chimpanzees seem to know they are sick since they resort to self-medication when exhibiting signs and symptoms of disease. Also, they help those exhibiting physical and cognitive disability. Among hominins, awareness of consequences and implications of sickness and coping with them represented an important feature of human consciousness and a major factor in the origins of vaunted human abilities involving language, cognition, and culture as we know them. A philosophical examination of the early evolution of sickness and healing provides a window into an understanding of evolving human capacities such as self-awareness, awareness and implications of suffering, theory of mind, altruism, conceptual grasp of sickness and healing and morality.

## Introduction

Evolution of human mental capacities represents a recondite problem construed as speculative and beyond the reach of empirical research in behavioural sciences and the clinical sciences. Yet, the evolution of cognition, language, and culture as we understand these capacities falls squarely within the perimeter of *Mens Sana Monographs*’ concerns which, while centred on human behaviour and its problems, begs answers to larger questions including that of origins. The role that sickness and healing behaviours may have played in evolution of human mental capacities certainly falls within the purview of *Mens Sana Monographs* and further reinforces the relevance of this problem for deliberation among scientists, philosophers, and clinicians.

The topic of origins of mind and minding got started in earnest through the observations and insights of ethologists and biologists who framed the problem as involving forms of animal awareness, fixed action patterns, instinctive behaviour, adaptive behaviour, and the general organisation of behaviour including even culture. During the last few decades of the 20^th^ century, this area of scientific interest gained momentum, diversified, and flowered. Today, some of these ideas and others they spawned represent a major area of research and scholarship in biology, anthropology, and psychology (Thorpe, 1956; Tinbergen, 1963; Griffin, 1976 and 1992; Bonner, 1980; Bekoff, 2002). Developments in contemporary social evolutionary sciences such as Human Behavioural Ecology, Evolutionary Psychology, and even Dual Inheritance Theory either owe their birthing to the earlier ethologists and naturalists or have been influenced by their perspectives on behaviour.

In studies of primate cognition, in particular, questions about the nature of mind and minding and related ones involving self-awareness and self-consciousness are staple themes. They cover observations and experimental data involving chimpanzees (Goodall, 1986 and 1988; de Waal, 1996 and 2005), the antecedents and properties of morality and of moral minds (Hauser, 2006) and proceed onwards to examine the influence of language on self-awareness (Bickerton, 1990 and 1995; Macphail, 1998 and 2000), and the cultural origins of human cognition (Tomasello, 1999).

The frame of reference in studies involving primate psychology and cognition (Maestripieri, 2003; de Waal, 2001) involves ideas and principles of social evolutionary sciences. The latter exemplify the thesis of evolutionary continuity (from primates to *Homo sapiens*) involving aspects of cognition, behaviour, social organisation, and culture. The topic of *origins* of human forms of mind and minding, as we understand it, is thus a subtext in studies of cognition in higher primates. Higher primates are held to embody and best represent the ancestral models of human cognition. Undergirding such studies is the assumption and expectation that research on relevant topics can contribute to a better understanding of the organisation of the brain and cognitive capacities and behaviour of *H. sapiens*.

## Orientation and Rationale

The general question being examined in this presentation involves the relationship between disease, sickness, and medicine, on the one hand, and origins of human forms of mind and minding, on the other. The influence of disease on population genetics and physiological response patterns exemplify the fields of Evolutionary Medicine (Stearns and Koella, 2008) and Darwinian Medicine (Nesse and Williams, 1994), but here the focus is on morbid effects of disease as sickness construed as a holistic biopsychosocial entity (Engel, 1960 and 1977). The genes documenting the history and sources of disease in human populations and a focus on the inner physiological consequences of disease certainly are crucial to an evolutionary understanding of disease, but it should be recalled that disease enters the social spaces of groups of organisms through a small portal as sickness and the way the latter impacts on behaviour of organisms represents a problem area worthy of examination and research. Stated in general terms, analysis of the behavioural effects of disease in higher primates represents a problem area which is relevant to the question of the evolution and nature of human cognition and consciousness; and the conjunction of these topics sheds light on the cultural evolution of medicine as social institution.

The material discussed in this article centres around observations by medical ecologists of self-medication behaviours of higher apes and response of group mates to clinical manifestations of disease diagnosed by careful observation. Given the importance of disease as a factor of natural selection and the evolutionary significance of higher apes, behaviours surrounding morbidity effects of disease provide a stage on which one can consider evolving templates of human cognition. Two assumptions exemplify and motivate discussion: 1) An adaptive behavioural response to observable condition of disease involves mediation of complex brain/behaviour or body/mind relationships and 2) healing of self and healing of a group mate (i.e., self-healing and other healing) represent adaptive response patterns (conscious or non conscious; intentional or “instinctive” and innate), the function of which is to counteract an evolutionary costly condition of sickness. Self-healing raises question of self-awareness of sickness and other healing that of awareness of sickness of another, which encompass a theory of mind.

What is it that the higher apes do when sick to improve their health status, and what their behaviours might mean from a cognitive, cultural, and evolutionary standpoint, the focus of this paper, provide an opportunity to examine elementary features of thinking tied to natural selection for survival and fitness. The behaviour programmes responsible for such biological goals and functions represent evolutionary imperatives (Fabrega, 2002). This topic necessarily broaches on the question of related forms of cognition and behaviour among earlier varieties of genus *Homo* leading up to the advent of *H. sapiens*. Sickness and healing are behavioural universals and how these are understood and carried out represent the centre point of any system of medicine one may characterise as cultural (Fabrega, 1974, 1975, 1997, 2002, and 2009).

## Studying Origins of Mind and Minding

### Forms of consciousness

The study of consciousness is broad and complicated and only a brief schema is given attention to in this paper. Damasio (1994 and 1999) points out that an animal’s awareness depends on *core consciousness*. This form of readiness to react and act (i.e., awareness and responsiveness) is correlated with and rooted in visceral somatic processes, functions, and responses centred in the midbrain and hypothalamus, which correlate with recruitment of the reticular activating system with diverse correlates (e.g., heart rate, blood pressure, respiration, endocrine responses). Although not elaborated by him, one can presume that from a cognitive standpoint, the so called “core consciousness” involves (an organism’s) sense of self for or about a moment in time - what is happening now and where. Core consciousness does not encompass access to (i.e., awareness of or knowledge of) conventional memory, working memory, reasoning or language. Its scope is “what is on line” at the centre of the attention regulation system, with no sense or awareness of the future and a minimalist sense of the past and no sense of authorship or self.

Damasio’s more complex and elaborated form of consciousness, *extended consciousness*, involves levels and grades of awareness of self and situation, forms of knowledge that evolve over an individual’s lifetime. Extended consciousness attains its highest levels in humans who exhibit a sense or understanding of self or autonoeisis (Tulving, 2005), sense of community or group, and a sense of a historical timeline which is based on a capacity to mentally travel into a remembered past and imagined future. *Autobiographical memory* and *autobiographical identity* are correlates of extended consciousness in humans – the self’s experience and awareness of a past and present situation. For example, in certain types of complex partial seizures, the individuals affected are awake and alert, seemingly attentive to surroundings, objects and happenings, and able to respond to and behave in relation to what is taking place. However, they do not exhibit a sense of authorship or ownership of a being with a sense of historical identity; that is to say, they are deprived of a sense of self as *comprehensively knowing* what it is they are witnessing and literally doing.

### Additional considerations

The problem of consciousness can be viewed in a more extended neuropsychological information-handling format. It encompasses thinking, for example, perception (e.g., seeing, hearing, smelling, touching), working memory and executive functions (e.g., image formation, retrieval of memories, comparison, selection) and action (e.g., doing, holding from doing).

The neuropsychology of mind and minding is layered onto (and can be limited to) capacity *of thinking without words* which brings the topic into the arena of primate cognition, namely, forms of thinking and acting of beings without language as we understand this (Bermudez, 2003; MacPhail, 1998 and 2000). There are three domains involving experience and behaviour to which the *thinking-without-words paradigm* is applicable. These are non-linguistic thought of animals, human infants (i.e., pre-linguistic humans), and hominins that followed the pongid/hominid split of 5-6 million years ago. Thinking without words is an appropriate paradigm for the analysis pursued in this article: the non linguistic thinking of chimpanzees framed in terms of conditions of disease, sickness, and motivated responses (conscious or non conscious) for purposes of understanding evolution of human cognition and behaviour, and of medicine.

### A schema linking body, mind, minding, disease, and medicine

Animals exhibit awareness and consciousness of their environment. Mating and parenting, securing food resources, responses to injury and noxious toxins, overcoming challenges of predators, and appetitive behaviours favouring ingestion of nutrients that maintain health and promote fitness are examples of elemental parameters of animal life that reflect adaptive behaviour and forms of minding or cognition. They represent phenomena that exemplify evolutionary imperatives; in other words, factors intrinsic to the organisation and timing of milestones involving life history theory (LFT) are the product of natural selection.

Evolutionary foundations of human cognition raise questions about the nature of consciousness and awareness; in particular, self and other awareness, past experience and learning, working memory and its retrieval of declarative and nondeclarative memory stores, and executive problem solving. In complex and social animals like mammals, mediation between sensation and perception through decision making and then motor response have been construed as forms of animal thinking, awareness, and culture (Griffin, 1976 and 1992; Bonner, 1980; Bekoff, 2002; De Waal, 1996 and 2005).

Elements of primate psychology bear directly on the question of adaptive behaviour in the context of disease. Responses to the effects of disease which contribute to improved health suggest that motivation and intention are representing preconditions, if not cornerstones of medicine, considered as a set of meaningful social practices designed to alleviate the costs of disease. Indeed, the origins of sickness and healing and the evolution of medicine and psychiatry as social institutions represent chapters of conditioned by higher primate behaviour responses surrounding occurrences of disease, the central topic of this article (Fabrega, 1997, 2002, and 2009).

## Sickness and Self-healing in Chimpanzees

### Prolegomenon: Health maintenance and the natural biology of animals

Disease and sickness compromise utilisation of resources necessary for maintaining life and meeting milestones of life history theory (LHT). Evolution of behaviours that promote health and prevent disease can be construed as medically relevant although they explicitly may not counteract the effects of disease. Behaviours governing adaptive use and avoidance of misuse of environmental products are to be expected in natural populations of animals (Engel, 2002). Many items consumed primarily for their nutritional value can have health-promoting and medicinal properties as well. Indeed, the line separating food value or protective action and actual medicinal medical value can be difficult to draw (Johns, 1980; Fabrega, 1997).

Examined in evolutionary context, health-promoting behaviours in higher primates involves linking their local (i.e., proximal) motivations and circumstances with remote or ancestral (i.e., distal) evolutionary processes as causes (Tinbergen, 1963). While general evolutionary imperatives of such ultimate causes of adaptive behaviours are understandable, what they translate into and how they actually come into play in proximal circumstances in individual organisms is not clear and contested. Behaviours responsible for avoiding disease, and the (learning) mechanisms that implement this, have been explained as innate and “hardwired”, physiologically conditioned responses based on previous aversive experiences (e.g., conditioning of an door, place, or taste which later produces noxious experience), observation of kin or non-genetic adults, or voluntary and directed based on trial and error learning (i.e., avoidance of disease) (Garcia and Koelling, 1966; Johns, 1980; Hart, 1988 and 1990; Lozano, 1998; Revusky, 1984; Rozin *et al*, 2000; Engel, 2002).

### Disease and sickness in chimpanzees: General considerations

Infection and re-infection by nematodes and other parasites can produce acute signs of symptoms of sickness in chimpanzees. Observations in open fields of Africa have provided opportunities for primatologists to diagnose conditions of disease or sickness among chimpanzees and infer the possible significance of behaviours that correlate with it, in particular, behaviours that may represent adaptive or maladaptive responses. Recent studies of diagnosis of conditions of disease in chimpanzees are based on observations of changes in physiology (e.g., “signs” of disease such as diarrhoea, vomiting, skin lesions, presence of parasites in stools, changes in the condition of the fur) and demeanour/behaviour (e.g., lassitude, diminished level of activity, motor coordination or ambulation difficulties, level of interest and participation in social activities).

Some chimpanzees when they are sick select and ingest leaves which are not part of their regular diet. The ingestion involves plants and plant products which seem to be keyed to the condition of sickness, which were not only suggested by observation but also corroborated by examination of material egested in stools. The ingested material includes a) chewing and swallowing plant products, the chemicals of which are harmful to infective agents and b) by swallowing but not chewing abrasive leaves of other plants which appear in the faeces of chimpanzees undigested and coated with parasites (Huffman, 1997, 2001, 2005, and 2006; Alados and Huffman, 2000). Viewed in anthropomorphic terms, it is as though higher apes embodied knowledge, genetic and/or learned, which they exhibited when sick and on the basis of which they engaged in behaviours (ordinarily not resorted to when not sick) which may have promoted health in prior sickness conditions.

### Self-healing behaviours as a chimpanzee behavioural tradition

A factor that makes self-healing in chimpanzees relevant for an understanding of the cultural organisation and evolution of sickness/healing behaviours is that similar patterns of self-healing are exhibited by many members of different groups of related chimpanzees. Self-healing behaviours are found in varied populations of the same species that are geographically and ecologically isolated from one another. However, not all self-healing behavioural ensembles are the same in geographically or ecologically distinct populations of chimpanzees; for example, different plants are used. Hence, behaviours of self-healing in any two populations displaying such differences are unlikely to have been transmitted from one (more or less isolated) population to another. The details of self-healing behaviour and type of species of plant that are selected differ across populations, sib species, and sympatric species of chimpanzees.

Researchers have identified differences in social behaviour routines in separate communities of chimpanzees and describe these as *behavioural traditions* which are construed as analogues of culture (McGrew, 2004). Chimpanzees show many behavioural traditions; for example, stone handling, tool making and use, and sexual signalling. Patterns in the use of medicinal substances during sickness also differ across groups. Huffman opines that the distribution of self-healing behaviour across chimpanzee communities represents differences in behavioural traditions. This would make the architecture of self-healing behaviours in chimpanzees part of their emergent or proto “culture” of medicine in analogy to how in *H. sapiens* ideas and beliefs about sickness and healing constitute a unit or item of information of their medical culture or ethnomedicine.

While behavioural traditions of self-healing indicate differences across populations of chimpanzees, its wide distribution and function beg a formulation of its evolutionary biology. It is possible that the self-healing routine involves an innate bio-behavioural programme, based on genetic and epigenetic inheritance systems, which is further sharpened through observation learning and ecologically tweaked in different ways, hence also part of a behavioural inheritance system (Jablonka and Lamb, 2005). Self-healing would then represent a behavioural routine that originated in and was transmitted across members of the species which at an earlier date formed connected populations. Subsequent ecological and geologic changes producing isolation between chimpanzee populations or subspecies may have then been caused via a sort of cultural drift conforming to the current pattern of separate traditions. On the other hand, self-healing traditions may simply represent fully emergent learned routines in separate populations. In other words, explanation of contemporary differences in tradition of self-healing may be the consequence of intrinsic developments within populations and subspecies that took place either before or after ecological separation. The ontology and epistemology of sickness and healing behaviours are taken up later in this paper.

### Acquisition of self-healing behaviours

It is not clear how adult chimpanzees come to acquire self-healing behaviours from group mates in the first place. The possibilities include pure individual self-learning through trial and error and the formation of selective associations (e.g., of inherited predispositions) or conditioned response patterns. Alternatively, self-healing may be a product of social learning (e.g., involving imitation, social facilitation, social enhancement, a resultant of emotional communication or empathy, and social reinforcement). According to Huffman, it appears that infants learn about self-healing through observation of their sick mothers ingesting the plants. They take a turn at tasting its bitter flavour which, however, they (i.e., the infants) do not at this time ingest. One possibility, which is discussed later, is that chimpanzees may be involved in social learning of self-healing at a very early age and probably reinforced thereafter.

Acquisition of knowledge of a particular plant’s medicinal value and of its relevance as a disease-counteracting routine involves several possibilities. If an individual learning pure and simple is the basis for acquisition of self-healing, then trial and error and resorting to adventitious products of the ecology when the organism is sick would commence the learning process, and one presumes the conditioning reinforcement comes later if and when a particular plant product or some other routine relieves signs and symptoms of sickness (Garcia and Koelling, 1966; Garcia *et al*., 1974). Chimpanzees and monkeys show many behaviours that qualify as self-medication (e.g., geophagy, fur rubbing) which might conform to an individual learning routine.

A social learning routine is more complex. The first step is individual’s observation of a diseased sibling, most likely its mother, or non-genetic adult resort to self-medication via plant ingestion. The second step involves subsequent use by the observer of the plant used by the teacher or modeller in a context of its own condition of sickness. As mentioned earlier, the same form of learning may involve other varieties of self-healing (e.g., geophagy, picking ectoparasites while grooming). They provide the individual with a baseline for future trial and error learning and social learning. On the other hand, Huffman suggests that acquisition of a self-healing routine might be the outcome of a “one shot” selective association going from sickness to healing. The learning routines of sickness and healing are taken up in more detail in what follows.

### Motivations for self-healing routines

#### General considerations

To examine chimpanzee self-healing during sickness as a test case for the study of evolution of mind and minding, one can start with the assumption that the behaviour in question does not represent the product of a conscious, conceptual and hence wilful decision. One essentially puts aside intervening variables or constructs such as self-consciousness, self-regulation, mindfulness, and intentionality. Several questions can be raised when one construes self-healing as per ingestion of plants or fur rubbing as an “associative ensemble” in a non-human primate whose cognitive capacity is in question. More directly: How does one explain the origin of behaviours which appear to represent an adaptive response to stimuli about an individual’s own changed state of sickness? Is directed or trial and error learning about self-healing the result of adventitious learning via conditioning following inspection of physical characteristics of plants found in the ecology? If this is the case, then what does the learned information consist of? And, furthermore, how does the learned information about sickness and healing come into play so as to produce later acts of self-healing?

### Individual learning

Leaving a concept, like intentionality, out of the picture and addressing individual trial and error learning, one is led to consider behavioural responses to sickness in a purely physiological sense, namely, as involving mechanisms of conditioned association between sickness and self-healing viewed not just as behaviours but in terms of internal systems. Visceral somatic physiological sensations (or “emergent” psycho-physiological perceptions) tied to sickness (i.e., physiological correlates of signs and symptoms) represent endogenous signals launched by a sickness condition which influence brain centres controlling sensation, perception, and action. Brain processes and networks come to encode the information handling that constitutes a routine of sickness and healing. These include, in addition to changes in the internal environment (i.e., the physiology underlying sickness), information about properties of plant and related ecological features, and the physiological consequences of acts of self-medication. Sheer physical properties or “intuitively” recognised characteristics of sickness and of faunal elements of the habitat provide information to the sick chimpanzee (conditioned or cueing stimuli) which signal reinforcement of a response programme of self-medication that when activated relieves signs and symptoms following selection of a particular plant or plant leaf. A scenario or routine such as this appears to correspond to what has been described as “inherited predispositions” as formulated by Revusky (1984; see also Hart, 1988 and 1990, Lozano, 1998, Huffman, 1997, 2002, 2005, and 2006, and Fabrega, 1974, 1975, 1997).

The process of learning to self-heal in the event of sickness can be likened to the obverse or reverse of the conditioning routine involving avoidance of chemicals that cause disease. In this instance, the organism, while in a state of relative health, avoids ingestion of a chemical that in a prior testing situation caused a condition of sickness (Garcia and Koelling, 1966; Garcia *et al*., 1974). This falls under the category described earlier of sickness avoidance as a motivated adaptive behaviour response. The process of self-healing involves motivation to counteract a condition of sickness (Revusky, 1984; Lozano, 1998; Rozin *et al*., 2000). While unclear, it would be surprising if a self-healing routine is not reinforced socially by repeated exposure to sickness/healing behaviour in others, and/or transmitted from adult to adult in the first place (as evolutionary anthropologists are likely to suggest) which involves social learning (discussed below). Indeed, one can propose mediation of mirror neurons linking observation of another’s self-healing routine while sick with acquired, neurologically enculturated routines in response to sickness (Rizzolati and Craighero, 2004; Rizzolati and Sinigaglia, 2008).

### Social learning

Moving beyond the pure trial and error conditioned association pattern of learning, a non-human primate’s routine of self-healing could represent a socially learned sequence of behaviour which is non-conscious. This formulation of self-medication behaviour in a higher primate does not negate the earlier explanation of pure individual trial and error learning based on associative predisposition (“mindless”) as an explanation: rather, it builds on, elaborates upon, and is not inconsistent with the earlier formulation. However, a socially learned yet non-conscious, non-intentional basis for a self-healing routine also raises several questions. How did the individual acquire the behaviour from a group mate in the first place? How does an individual who is sick learn to use a hypothetical socially learned routine at an appropriate time (i.e., when it is sick)? What about (or how did) the teacher of this behaviour signal or cue to the learner that the behaviour of plant selection/ingestion (i.e., self-healing) is “naturally appropriate” or “adaptive” in conditions of sickness?

These and related questions circle around the motivation for or stimuli-triggers which in relation to a social learning paradigm activate what may represent a natural, innate predisposition to avoid and counteract effects of disease in a higher primate who has not evolved self-awareness, self-consciousness, sense of intentionality, and awareness of changes in its state of being. It would seem that for this socially learned routine to get installed as a behavioural response pattern of a learner, a cause/effect connection has to be established between observation and perception that a putative teacher (or modeller) exhibits sickness behaviour (or signals it) and resorts to the ingestion of plant material which is specially selected (i.e., not ordinarily ingested as part of routine diet preferences). As a consequence of this perception, the learner comes to ingest the same or related plant material when it itself is sick.

Knowledge that a group mate has information that is functional, adaptive, and useful in relation to sickness presupposes complexities of mind and minding not currently attributed to chimpanzees. One would presume that part of the socially learned sickness/self-healing routine includes the learner’s observation that ingestion by the teacher was followed by amelioration of signs and symptoms (or change in behaviour) towards “normal” baseline. In this scenario, social learning could proceed in terms of factors that primatologists construe as local enhancement, emulation and or imitation (the latter, very unlikely).

In summary, when a routine of behaviour is acquired through nonconscious social learning, three factors get conjoined: a) a learner’s perception that a group mate (most likely genetically related) is exhibiting sickness which is exemplified in a changed routine of its behaviour (e.g., signalling through the signs/symptoms of sickness); b) the learner comes to learn from observing the teacher that ingestion of plant is appropriate mainly under special conditions (when sickness supervenes); and c) the learner when sick is able to somehow connect or associate its present state (of sickness) with that of (an episodic memory about) an observation involving teacher when it was sick, from which the learner acquired information about plant ingestion routine in the first place (i.e., when teacher exhibited signs and symptoms of sickness).

Parenthetically, emphasis is given to parental figures or kinfolk in the modeller/teacher compared to learner roles because origins of sickness and healing routines in a social learning context bring into the picture genetic implications of aiding another individual (who functions as a recipient or benefactor) at a potential cost to the giver or provider. This consideration exemplifies the biological problem of altruism and morality (Alexander, 1987). Stated baldly, to provide adaptive information confers advantages and benefits to a recipient of an act or information about sickness and healing at a cost to the provider, and the logic of this in a world dominated by evolutionary imperatives of competition and survival requires explanation. The theory of inclusive fitness stipulates exchange is likely among genetic relatives (Hamilton, 1964). Alternatively, the theory of reciprocal altruism (Trivers, 1971) stipulates that the costly giving of benefits to another is functional and adaptive providing the dynamics of exchange are part of a sequence which involves reciprocation in future circumstances of need and giving of benefits. Discussion of these issues is beyond the purview of this presentation.

### Effects of self-healing

Discussion thus far suggests behavioural contingencies for the social learning of non-wilful, non-deliberative actions involving a self-medication routine. It presupposes an origin to the sequence. This presumably is the result of conditioning of improvement following ingestion of a plant. This would represent knowledge from mere adventitious association acquired through classical conditioning or positive reinforcement. As suggested earlier, this process is analogous to (i.e., similar in motivational and reinforcement contingencies) but the reverse of the negative reinforcement involving the evolution of bitter and acquisition of toxiphobia (see Garcia and Koelling, 1966; Garcia *et al*., 1974). In the case of chimpanzees, conditioning to a future state of improvement is consistent with their ability to cognise forwardly up to 18 or so hours (as reflected in the ability to save tools for future use).

To go beyond the point of origins of self-healing as a purely associative pre disposition, necessarily elicited by and/or tweaked by ecological stimuli, and frame the behaviours in question as a socially learned routine, one can say that a chimpanzee observes a model ingest plant material when sick and learns that the plant ingested was restorative or ameliorative (a self-healing act) for the model or teacher. Once the routine is incorporated, it becomes habitual as the learner experiences physiological or psycho-physiological improvement (i.e., reinforcement). As indicated earlier, to propose that a higher primate teacher engages in pedagogy (towards the learner) as per the self-medication routine is to ascribe to it a higher form of cognition which studies in primate cognition do not support.

## Other Healing in Chimpanzees: Responding to a Sick Group Mate

While self-healing has received concerted observation and research, providing support, care, and medical healing to a group mate (termed here other healing) has not. The latter rests on anecdotal information of primatologists and consists of observations of help provided to handicapped group mates, licking and cleaning of wounds, and general response patterns involving supportive and comforting behaviours (reviewed in Fabrega, 2002). Other healing, it should be noted, raises the question of an organism’s point of view regarding a group mate’s state, condition, or readiness state. Other awareness is often described as theory of mind (i.e., an organism’s understanding or insight about another’s mental contents).

Several constructs not easily separated from one another are used to formulate how primates understand and react to the plight of others (Silk, 2007). In addition to theory of mind, these include, for example, *emotional contagion* (instinctive reaction to another’s distress), *empathy* (ability to understand and respond to distress in another and perhaps appreciate distinction between other and self) and *sympathy* (feelings of actual concern for welfare of another). Social emotions such as these not only influence but are inherent in cognitive representations and actions related to sickness of a group mate (which will be termed here *other healing*).

Empirical support for other healing in natural communities of primates is not voluminous. Some observation studies of primates, including chimpanzees and even monkeys, have demonstrated that in some instances they respond with support of conspecifics (also called “group mates”), who are victims of aggression, or exhibit behaviours involving sickness, trauma, and disability. Best examples involve licking and cleaning of wounds, attempts to cover or stop bleeding, responses towards handicapped individuals of very young age, and the worried concern and protectiveness towards infants (reviewed in Fabrega, 1997, 2002 and 2006).

There is controversy about whether sickness represents a *special context* for social behaviours involving care of a group mate who is sick and suffering. Sickness/healing behaviours may represent a token or variety of more general pro-social, altruistic behaviours (which may function as biological roots of morality - mentioned earlier) which merely happen to be exhibited in this (i.e., medically relevant) context (as compared to others with similar functional implications). The observations and analyses of DeWaal (1996) (e.g., consolation, empathy, sympathy, and conflict negotiation) and material discussed and critically examined by peer commentary in Preston and DeWaal’s review article (2001) also suggest generic indices of intentional caring (and in this sense, “healing” as comforting) behaviour. With respect to sickness/healing per se, in addition to the material summarised in Fabrega (1997 and 2000), there is the report by Huffman and Seifu (1989) on females caring for infants of sick mothers, who cite observations involving more directed support and concern for the mother’s suffering.

However, there is sparse information about relevant cognitions devolving from controlled experiments or counteracting material on higher primates which suggest a *generic empathy and sympathy* towards plight of others. Furthermore, there are no hard data that would clinch a proposition about other healing: scanty results of experiments involving interpretation of behaviour that might be taken to support understanding of another’s plight of sickness and providing aid and/or rewards to others in conditions of distress manufactured through experimental protocols (see Silk, 2007, for review).

In general and on theoretical grounds, sickness and both self and other healing in chimpanzees imply experience of embodiment and sharing of representational content involving cognition, visible external somatic changes, internal neuro-vegetative changes, and social behaviour associated with sickness. The behavioural link between self and other healing, like the connection between inner visceral responses in sickness and the perception of distress in another, could be based on a network of mirror neurons in the frontal lobe of the brain (Brothers, 1990 and 1997; Preston and DeWaal, 2001; Rizzolati and Craighero, 2004; Rizzolati and Sinigaglia, 2008). An individual’s cognitive awareness of and response to internal changes in physiology stemming from sickness (giving rise to a need for self-healing) along with responses triggered by observation of external manifestations of sickness observed in and/or communicated by another’s plight (e.g., involving facial, vocal, or postural cues, distress signals, evidence of gastrointestinal or respiratory) are elements that can be construed to constitute the biopsychosocial wholeness and neuroscience underpinnings of sickness and self and other healing. They provide a test case for discussion of origins of mind and minding; more specifically, of higher forms of cognition in an area of social life of basic importance to survival and fitness.

In general, whether animals, including higher primates, exhibit, and can be presumed to exemplify, a capacity for empathy, and especially sympathy, in the context of sickness, or generically in the contexts of altruism, raise questions that have empirical and philosophical implications and about which there is much contestation. The topic is implicit in academic thought pertaining to animal awareness and comparative psychology and feeds into the question of the innateness of human experience and behaviour in matters of sickness and healing, morality, and altruism (Bonner, 1980; Bradshaw and Sapolsky, 2006; Bradshaw and Schore, 2007; Griffin, 1981 and 1992; Brothers, 1990; Engel, 2002; Preston and DeWaal, 2001; Zahn-Waxler, 2002). As in the case of experimental investigation of health maintenance as a biological problem in higher primates (discussed earlier), the study of responses to sickness either via self-healing compared to other healing, perhaps involving an actual communication of sickness from a victim of disease morbidity to a group mate who may or not exhibit sickness, has not fallen within the envelope of behavioural ecology paradigms.

It is certainly contestable whether “other-healing” behaviours in animals and even higher primates represent *conscious awareness* of, concern for, and directed response to the plight of disabled group mates (like self-healing might regarding awareness of sickness in self). Yet, on the other hand, Troisi and McGuire (1991) suggest that sickness can appear to function very much as a socially significant object. They report that non-human primates can dissimulate a condition of sickness and/or handicap so as to tactically deceive another. Thus, they suggest awareness and meaning of sickness as a social or psychological condition per se, and this would seem to be a step beyond an intuitive heuristic or proto concept since individuals seem to feign sickness for self-advantage. Put differently, “medical malingering” seems to imply awareness of what sickness means in a social context and hence a capacity for sharing a code, however elusive its meaning and scope (e.g., an ability to understand and use behaviour of sickness as a symbol). This line of thought would imply that already in higher primates, and presumably in the last common ancestor (LCA) of humans and higher primates, sickness related behaviours and meanings represented an emergent *culture of medicine*. On the other hand, the anecdotal report of Troisi and McGuire (Troisi and McGuire, 1990) might represent simple individual coding, a resultant of an individual’s non-conscious, conditioned association of sickness and what it (i.e., sickness) elicits behaviourally and not a truly self-conscious understanding and even less an emergent socially shared system of codes (Chase, 2006) about what sickness means which motivates and emplaces sickness in a conceptual structure.

## General Comment on Other Healing

The preceding line of thought can be framed in anthropomorphic terms. In association with and as a consequence of a condition of sickness in a particular social group (e.g., a higher primate, hominin, representative of genus *Homo*, infant or adult *H. sapiens*), the individual victim is motivated to directly heal itself. It might be its intention to communicate its plight to an audience perceived as of potential help (e.g., parental figure, sibling, group mate) for purposes of eliciting other healing. Finally, a member of the audience may intentionally provide other healing to the victim.

In a related scenario, upon perceiving that a victim is diseased, a group mate may be motivated to provide other healing regardless of whether it is communicated intentionally by a victim. This could be a consequence of (a) the group mate’s *conceptual (i.e., conscious) understanding of sickness as a changed biopsychosocially altered state* (i.e., of self or of an individual) or (b) a consequence of a *visceral somatic intuition* about sickness that another is sick and suffering (a so-called natural, intuitive heuristic; Boyer and Barrett, 2005) mediated, for example, as neurological mirror-neuron response pattern and not necessarily conscious. The group mate, as it were, has innate intuition in relation to occurrence of sickness; alternatively, group mate has intuitively learned to match what it perceives in the victim (i.e., presently) with an episodic recall of sickness in itself (i.e., a past illness of self). The victim, in other words, has learned what to do for a victim who is perceived as being (i.e., who happens to me modelling sickness) based on previous experience of sickness (Fabrega, 1997).

Sickness/healing in chimpanzees can exemplify behaviours that are non-conscious/innate and conditioned compared to conscious/intentional. Either scenario suggests that in higher apes, evolution has productively mined the biological information inherent in biopsychosocial conditions of sickness. It has sculpted adaptive behaviour patterns in response to occurrences of sickness, thereby providing individuals of highly social species, especially higher apes, with a behavioural template or idiom for adaptively understanding, responding to, and communicating information regarding its fitness plight as per imperatives tied to evolutionary and Life History Theory (LHT) (Fabrega, 1975; 1997).

## Summary and Conclusions

Philosophers and theoretical and philosophical psychologists tend to construe mind and the activity of thought and action which a “mind” is said to authorise (i.e., minding) in abstract, analytical, and logical terms. Except for brief examples which illustrate and clarify examples of constructs, principles, and propositions, philosophical analyses of “mind” are generally removed from the plane of human history and thus in many ways are construed as timeless and universal. In this paper, “mind” and “minding” have been not only forced down into “real, on the ground” social contexts, but also presumed to have an origin in human history; specifically, human biological (i.e., evolutionary) history.

The problem of origins is of central concern to the traditions which frame the argument and positions taken in the article, namely, evolutionary psychology, biological, evolutionary anthropology, primate cognition, and evolutionary and Darwinian medicine. Such fields of inquiry represent areas of empirical investigation that are dynamic and expanding with claims, counter claims, and insights which challenge the usual dualist notions involving not only animal and human but, more generally, between experience and behaviour, thought and language, mind and body, culture and nature, ideational/conceptual and physical/organic, and naturalism and supernaturalism. The paper has sought to locate early manifestations of mind and minding in activities of higher primate (and, by extension, hominin) groups; and in a domain that is universally disvalued, raises concerns, and gives rise to a need for corrective action (i.e., sickness and healing) classically thought of as conscious and intentional (Fabrega, 1974, 1975, and 1997).

One can reasonably propose that behaviours of higher apes in the context of disease bring into focus and perhaps exemplify earliest manifestation of an intentional and conceptual *understanding of and response* to changed, biologically undesirable biopsychosocial state of the self or person (i.e., awareness of signs and symptoms of disease). When writ large, such a formulation translates as the earliest instantiation of a cultural approach to medicine (e.g., a proto-medicine). More specifically, given their relevance as strategic models of LCA of man and apes, higher apes’ behaviours in the context of sickness can be likened to the earliest phase of evolution of ethnomedicine. The latter is construed as involving the cultural study of ideas, beliefs, and practices about sickness and healing which enable a directed, conceptual, and practical way of coping with the evolutionary hardships of disease (Fabrega, 1975).

Chimpanzee behaviours involving self-healing suggest that conditions of disease or sickness are things that an individual, if it were self-conscious and could tell us about, does not want. It exemplifies the dictum that a prime feature of sickness and disease is that it gives rise to a need for corrected action at the individual and social cultural level (Fabrega, 1974). When complemented with *other healing*, which takes place when an individual provides support, care, and healing to a group mate who exhibits sickness, one completes the primitive or elemental starting point of medicine considered as meaning filled social behaviour (Fabrega, 1997).

A group whose members heal themselves when they exhibit morbid conditions of disease (i.e., sickness) and heal others of the group when they exhibit sickness exemplifies a necessary behavioural template of ethnomedicine. If self- and other-sickness/healing behaviours of members of a group are conscious, intentional, and conceptual, then this qualifies as forming a foundational *H. sapiens* exemplar of ethnomedicine. Given the neurocognitive organisation of experience and behaviour and the evolutionary (biological and cultural) significance of disease and sickness, one can surmise that members of the group in question have acquired a deliberative, practical orientation towards existential (e.g., emotional, affective) and fitness (e.g., reproductive costs) implications of disease. The exemplar can be equated with or at least sets the starting point for an ethnomedicine, namely, the cultural patterning of sickness and healing which amounts to a social institution. Stated differently, complementarity of self and other healing presupposes some knowledge, awareness, and adaptive responsiveness about the significance of sickness of disease to orderly, adaptive functioning which can be formulated as basic elements of a cultural system of medicine or ethnomedicine.

**Figure 1:**
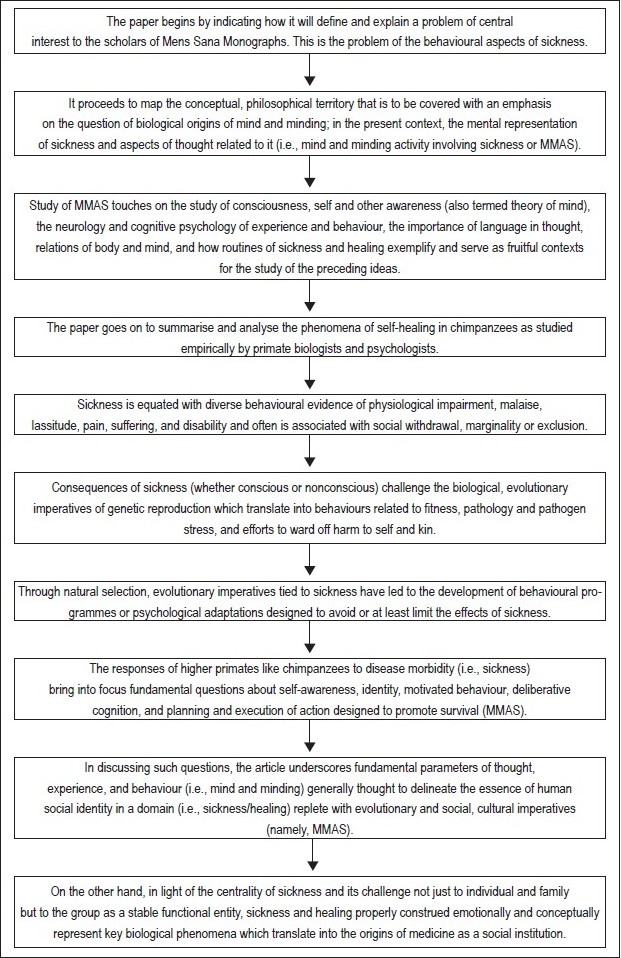
Flowchart of paper

A conservative position one can take is that chimpanzees do not mentally entertain (i.e., exhibit) *concepts* of self, self-healing, and especially, other healing. This implies that a conceptual representation of sickness/healing was not a component of thought exhibited in the LCA. Such concepts evolved later. Following the pongid/hominid divide and certainly with the advent of genus *Homo*, sickness and healing behaviour and the intuitive heuristic domain which was based on it can be presumed to be relevant to, and/or embed in, other areas of cognition and social and political life. Emergent awareness and more conscious use of social emotions (e.g., empathy, sympathy) generally and of the implications of sickness more specifically is one scenario for early evolution of medicine. Figure 1 summarizes the flow of the argument presented in this article.

Another consideration involves the influence of morality as a factor in social life, namely, *moral emotions and motivations*. The latter could represent a development linked to evolution language, culture and cognition as we understand it. Alternatively, the influence of morality in social relations might stem from, or be part of, intuitive heuristics that constitute the foundation of sociality; to have involved, for example, basic emotional understanding and behavioural dispositions that provide the glue for group formation. In this scenario, sickness and healing ensemble may be viewed as an “exaptation” of more fundamental psychological adaptations (Gould and Vrba, 1982; Gould, 1991; De Waal, 1996; Preston and DeWaal, 2002; Hurford, 2007; Hauser, 2006).

Empirical observations discussed in the article indicate that chimpanzees engage in self-medication behaviours when they are sick; and there is evidence that it is restorative of health. When unpacked, the “facts” of chimpanzee self-medication enable probing essential features pertaining to consciousness, self and other understanding, and motivated behaviour, all of which are of central interest to readers of *Mens Sana Monographs*. In addition, when aspects of mind and minding can be inferred from actions taken in the context of sickness and healing, they incorporate and correspond not only to questions about the origins language, cognition, and culture as we understand such phenomena, but also to the origins of a social institution of medicine construed here as an ethnomedicine. The latter construct refers to a people’s or group’s forging of a self-conscious, intentional, and conceptual understanding of the universal biological and cultural problems of sickness, suffering, longevity, and death; all of which have compelled human attention, thought, deliberation, explanation, development of nomenclatures, classifications schemes, knowledge structures, and action patterns.

### Take home message

This paper reviews and critically discusses diverse literatures in the behavioural and social evolutionary sciences towards a goal of persuading *Mens Sana Monographs* participants and readers that a) origin of thought (mind and minding) during human biological evolution represents an important theoretical problem begging analytical scholarly work and b) sickness and healing behaviours during human biological evolution represent a fruitful domain in which to study it.

## Questions That This Paper Raises

### A preface to research questions raised by the article

This article raises questions about the origins of fundamental human capacities (i.e., cognition, language, and culture) which correlate with a universally disvalued and motivationally salient domain (which can be conscious or nonconscious) of organisms, namely sickness. It begs logical analysis and empirical study of morbidity of disease (i.e., sickness related phenomena) in *social animals*. Because of their strategic relevance to human evolution, *higher primates and especially chimpanzees* (generally regarded as models of LCA of man and apes) represent important objects of empirical study. The *sickness dynamics* of chimpanzees and other non-human primates need to be studied empirically in captive, confined, or free ranging populations. Such study consists of and enables understanding of mind and minding activity related to sickness (MMAS).

Empirically, the following topics about MMAS need clarification: 1) the sequence of *physical changes* involving behavioural disorganisation and malfunctions (e.g., alacrity and level of arousal, quickness and organisation of behaviours related to visceral or vegetative activity such as ingestion and excretion, skeletal motor activity and reactivity) which can be a) observed under natural conditions of sickness, or b) produced by introduction of quantifiable but reversible amounts of somatic stress (i.e., agents or procedures causing sickness); 2) effects produced by conditions and procedures of 1a and 1b on routines involving *social behavioural changes* of sickness (e.g., social engagement contra withdrawal, negotiation over social status and rank, competition, mating, food gathering and eating).

Clarification of origins of MMAS begs *theoretical study* of changes in capacities for cognition, language, and culture of species that followed the pongid/hominid split. Such capacities are currently a focus of studies in cognitive archaeology and evolutionary anthropology and have focussed on nature of consciousness, immediate memory, span of short term memory space, and executive functions as these come into play in social exchange and behaviour. Changes in cognition and behaviour among *Homo erectus* and *Homo neandertalensis* are the principal areas of focus in cognitive archaeology, given nature of physical remains in sites which indicate enlarged brain size, more complex tool making abilities, and complex management of space and planning of movements in space. The theoretical task is to focus on and derive specific parameters of behaviour relating to sickness and healing which changed during the course of human biological evolution; for example, ability to name and describe features of sickness, differentiation of types of sickness, development of support measures and practices related to healing, individual and social learning of medicinal agents, and accumulation of knowledge which embodies MMAS.

Information pertaining to these topics can be formalised and modelled mathematically and studied through principles of human behavioural ecology and dual inheritance, co-evolutionary theory. Scholars who participate in the theoretical study of mind through *Mens Sans Monographs* are especial candidates for the theoretical study of MMAS in higher primates and among hominin species that followed the pongid/hominid split.

### Based on this preface, the following questions become relevant for future work in the field:

1. What types of bodily signs and symptoms and what degree of level of severity of sickness give rise to self-medication in chimpanzees?
2. Do chimpanzees show behaviours towards sick group mates when the latter are sick? If they do, what determinants of sickness elicit other healing? And, does such other-healing behaviour differ in relation to age or social status of recipient (e.g., kinship, grooming and/or reciprocating partner, ally/competitor, or social distance of group member)?
3. In the early evolution of (ethno) medicine, what parameters of genus Homo cognition (e.g., intentional self-awareness, theory of mind, knowledge of self (i.e., autonoeisis) working memory capacity, executive functions, time travel, conceptual understanding of suffering, death, sickness/healing) were primary and necessary and which ones were secondary and contributory?
4. Among the earlier representatives of genus *Homo* (e.g., *H. neandertalensis*), during late phases of human biological evolution, what priority is likely to have been given to sickness/healing activities (e.g., compared to routine foraging, social interaction, friendship development)? How would the social status of victim of sickness (e.g., age, gender, kinship status, and reciprocating nonrelative) have influenced a group’s decision about sickness/healing compared to, for example, avoidance, social exclusion or abandonment?
5. Do representatives of contemporary small-scale people (e.g., hunter gatherers, semi-sedentary dwellers, part-time horticulturalists) construe and respond to behavioural breakdowns (i.e., conditions of psychiatric interest) as they do compared to sickness ensembles marked predominantly by (physically evident and public) bodily signs and symptoms?
6. Are syndromes of behaviour which exhibit loss of emotional control (e.g., aggression, social divisiveness, social confrontation) dealt with exclusively in terms of quasi legal, policing, and/or administrative social controls?
7. Are ideas and practices of sickness/healing (i.e., ethnomedicine) brought into play in (some) syndromes of emotional loss of control?
8. What social parameters of the person exhibiting social “miss behaviour” influence the progression, resolution, and/or outcome of social exchanges?

## About the Author

*Horacio Fabrega Jr. is a practicing clinical neuropsychiatrist who is Professor of Psychiatry and Anthropology at the University of Pittsburgh. His academic work spans the fields not only of clinical and descriptive psychiatry but also of ethnomedicine, medical anthropology, cultural psychiatry, and evolutionary psychiatry. He is the author of four books and over 150 articles in peer reviewed journals in these fields. He was Director of Medical Student Education Program at University of Pittsburgh School of Medicine for 15 years. For the last 10 or so years, his work has encompassed evolutionary psychology and psychiatry, involving theoretical study of the way conditions of psychiatric interest have changed during various phases of human biological and cultural evolution.*
